# Altered intrinsic local activity and cognitive dysfunction in HIV patients: A resting-state fMRI study

**DOI:** 10.1371/journal.pone.0207146

**Published:** 2018-11-29

**Authors:** Yunjin Bak, Sunyoung Jun, Jun Yong Choi, Youngjoon Lee, Seung-Koo Lee, Sanghoon Han, Na-Young Shin

**Affiliations:** 1 Department of Radiology, College of Medicine, The Catholic University of Korea, Seoul, Korea; 2 Department of Psychology, Yonsei University, Seoul, Korea; 3 Department of Internal Medicine and AIDS Research Institute, Yonsei University College of Medicine, Seoul, Korea; 4 Department of Medical Education, Yonsei University College of Medicine, Seoul, Korea; 5 Department of Radiology, Yonsei University College of Medicine, Seoul, Korea; University of Texas at Austin, UNITED STATES

## Abstract

**Purpose:**

To characterize resting-state brain activation patterns and investigate altered areas for cognitive decline in HIV patients.

**Methods:**

Twelve male HIV patients with intact cognition (HIV-IC), 10 with HIV-associated neurocognitive disorder (HAND), and 11 male healthy controls (HC) underwent resting-state functional MRI (rsfMRI). Three rsfMRI values, regional homogeneity (ReHo), amplitude of low-frequency fluctuation (ALFF), and fractional ALFF (fALFF) were calculated and compared between groups. Correlation analyses were performed between rsfMRI values and neuropsychological tests.

**Results:**

rsfMRI analyses revealed decreased rsfMRI values in the frontal areas, and increases in the posterior brain regions for both HIV-IC and HAND compared to HC. When directly compared to HIV-IC, HAND showed lower fALFF in the orbitofrontal cortex and higher ReHo in the primary sensorimotor area. Additionally, decreased orbitofrontal fALFF, increased sensorimotor ReHo, and a larger difference between the two values were highly correlated with decreased verbal memory and executive function in HIV patients.

**Conclusions:**

Regardless of cognitive status, altered local intrinsic activities were found in HIV patients. The orbitofrontal cortex and primary sensorimotor area were more disrupted in HAND relative to HIV-IC and correlated with behavioral performance, suggesting these areas are relevant to cognitive impairment in HIV patients.

## Introduction

HIV infection-induced cognitive impairment, known as HIV-associated neurocognitive disorder (HAND), can occur without involvement of other possible causes such as opportunistic disease or malignancy [1]. Although combined antiretroviral therapy has decreased the incidence of the severe form of HAND [2,3], the prevalence of HAND, especially its milder forms, is still substantially high [4,5]. Even the milder form of HAND can decrease quality of life, making precise diagnosis and timely intervention essential. No theory has been established to explain the underlying pathophysiology for HAND, despite this knowledge being crucial for diagnosing and treating HAND. Moreover, numerous neuropsychological tests are used to diagnose HAND, even though they are time-consuming.

Resting-state functional MRI (rsfMRI) could be a good solution for defining underlying pathophysiology for HAND. It can reveal brain regions showing altered intrinsic brain activation [6–8], which can be possible targets for further conclusive studies to establish pathophysiology with a short scan time. A few rsfMRI studies have revealed altered connectivity in intra- or inter-networks for the default mode, executive, salience, dorsal attention, and visual networks in HIV patients [6,7]. However, these studies mainly focused on the differences between HIV patients regardless of cognitive status and healthy control subjects (HCs). A recent study [9] also reported decreased functional connectivity in the salience and executive networks of HIV patients with HAND compared to controls, but they did not analyze the difference between HIV patients with and without HAND. Therefore, studies are needed that investigate the brain regions relevant to cognitive impairment in HIV by comparing HIV patients with and without HAND.

Recently, our group directly compared HIV patients with HAND and those with intact cognition (HIV-IC) using a seed-based analysis, and reported decreased resting-state functional connectivity (rsFC) in the frontal areas with a predefined precuneus seed in patients with HAND [8]. To clarify the exact region of altered connectivity between the two regions and to explore other brain regions not assessed in the previous study, we used regional homogeneity (ReHo), amplitude of low-frequency fluctuation (ALFF), and fractional ALFF (fALFF) analyses in this study, since these are data-driven methods and can be used to assess the state of each voxel in the whole brain. ReHo measures how similarly a given voxel activates compared with its neighboring voxels [10,11]. Therefore, ReHo reflects how well a given voxel is working as a member of a functional module in the resting-state brain. ALFF and fALFF are used to assess the power of resting-state-specific low-frequency brain activation in a given voxel [12]. Until now, no study has observed alterations in intrinsic brain activity in HIV patients according to cognitive status using these methods together. The regional information obtained with these data-driven methods might contribute to better understanding of the pathophysiology of HAND and, furthermore, help define future therapeutic targets in HAND.

Thus, the present study aimed to define altered brain regions for cognitive decline in HIV patients by comparing HIV patients with HAND with HIV-IC and HC using data-driven rsfMRI analytic methods.

## Materials and methods

### Study population and assessment of cognitive status

This prospective study was approved by the Institutional Review Boards of the Yonsei University Health System and written informed consent was obtained from all subjects. Twenty-four male HIV patients (11 with HAND and 13 with HIV-IC) who underwent rsfMRI and a set of standardized neuropsychological (NP) tests [5] within a one-month interval between April 2014 and September 2014 were recruited as described in detail in a previous study [8]. Patients with focal brain lesions or comorbid conditions (i.e., traumatic brain injury, central nervous system infection, psychotic disorder, significant substance use, color blindness, and hearing deficit) that could influence cognitive status or daily functioning were excluded according to guidelines used in previous studies [5,13]. Eleven seronegative male HCs without any clinical cognitive impairment or active neurological disorders who were recruited as controls for a different study [14] were also included as controls in this study (See S1 File).

Six cognitive domains were assessed in HIV patients using a set of NP tests, and the scores of some NP tests were either age-corrected scaled scores (ASS; mean = 10, standard deviation = 3) or age-corrected standardized scores (SS; mean = 100, standard deviation = 15): (1) speed of information processing (Korean version of Wechsler Adult Intelligence Scale [K-WAIS] digit symbol subtest (ASS) and trail-making test part A [TMT A]); (2) memory including learning and recall (Korean version of auditory verbal learning test [K-AVLT; ASS] and complex figure test [KCFT; ASS]); (3) abstraction/executive function (Wisconsin Card Sorting Test [WCST; SS], K-WAIS similarity subtest (ASS), and trail making test part B [TMT B]); (4) attention/working memory (K-WAIS digit span subtest; ASS); (5) sensory perception/motor skills (Grooved Pegboard Test); and (6) verbal/language (K-WAIS vocabulary subtest; ASS). TMT A, TMT B, and Grooved Pegboard Test scores denote the length of time in seconds spent to accomplish the given task, and higher scores indicate poor performance; for other tests, higher scores indicate better performance. Patients were classified as cognitively impaired as performing below 1 standard deviation (SD) from the demographically adjusted normative mean of published results with HIV-negative subjects [15–19]. The Frascati criteria [13] were used to diagnose HAND.

### Imaging protocols

All examinations was performed on a 3T MR scanner (Tim Trio, Siemens Healthcare, Erlangen, Germany). 2D T2-weighted images (WI), FLAIR, and T2*WI were performed to rule out the presence of focal brain lesions. Then, 3D T1WI, rsfMRI, and diffusion tensor imaging were conducted in that order. Among all the obtained data, we only reported rsfMRI data in the present study.

rsfMRI data were acquired using a gradient echo-planar pulse sequence. From each subject, 150 axial volume scans were obtained with the following parameters: TR = 3 sec, TE = 30 msec, FOV = 192 x 192 mm^2^, voxel size = 3 x 3 x 3 mm^3^, slice number = 50 (interleaved). Total acquisition time was 7 min 39 sec. We also obtained 3D T1-weighted images for coregistration and normalization of functional data with the following parameters: sagittal acquisition with TR = 1900 msec; TE = 2.52 msec; FOV = 256 × 256 mm^2^; voxel size = 1 × 1 × 1 mm^3^; flip angle 9°; slice number = 176; and total acquisition time 4 min 26 sec. Vacuum-molded cushions and soft pads were used to support the head and minimize head movement.

### rsfMRI data preprocessing

rsfMRI preprocessing and statistical analyses were performed in Matlab R2014a (The MathWorks Inc., Natick, MA using Data Processing Assistant for Resting-state fMRI (DPARSF, version 2.3; http://www.restfmri.net) and statistical parametric mapping software (SPM8, Wellcome Department of Imaging Neuroscience, London, U.K.). The first 4 volumes of each image were discarded for magnetization stabilization. Slice timing correction referenced to the middle slice and realignment were performed. The data were coregistered to subject’s anatomical scan and spatially normalized to the Montreal Neurological Institute (MNI) template, then resampled into 3 mm cubes. Images were smoothed using an 8-mm full-width, half-maximum (FWHM) isotropic Gaussian kernel, but in the ReHo analysis, the smoothing was performed after calculating ReHo because smoothing before ReHo potentially inflates the results [11]. Then, the time series of each voxel was temporally band-pass filtered (0.01~0.08Hz) to reduce effect of possible very low and high frequency noise, and de-trended to remove linear trend.

### rsfMRI data analyses

ReHo measures the degree of regional connectivity by computing Kendall’s coefficient of concordance (KCC), a non-parametric test for correlation, for the blood-oxygen-level dependent (BOLD) signal of a given voxel and its nearest 26 neighbors representing a three-dimensional cube around the given voxel [10,11].

$$ReHo=\frac{\sum {\left(R_{i}\right)}^{2}-n{\left(\bar{R}\right)}^{2}}{K2(n^{3}-n)/12})$$

k = number of voxels within each cluster, n = number of ranks, Ri = sum of rank at ith time point.

To reduce the global effects of variability across participants, the ReHo of each voxel was divided by the global mean ReHo value for each subject.

Unlike ReHo, ALFF is not associated with correlation, but with the power coherence of specific low-frequency oscillations, reflecting spontaneous brain activities [12]. The preprocessed time series were transformed to a frequency domain using fast Fourier Transform (FFT). Because the squared amplitude of a given frequency component of the original BOLD signal is proportional to the power of the given frequency, the square root of the power spectrum was computed at each frequency of the power spectrum and then averaged across 0.01 Hz– 0.08 Hz. This averaged square root was the ALFF. fALFF measures the ratio of the power spectrum of a given frequency range to that of the entire frequency range and fALFF analysis has shown improved sensitivity and specificity in detecting spontaneous brain activity compared to ALFF [20]. As done in ReHo, the ALFF or fALFF of each voxel was divided by the global mean ALFF or fALFF, respectively, for each subject for standardization.

We identified group differences in ReHo, ALFF, and fALFF using one-way analysis of variance (ANOVA) and post-hoc analysis using ANOVA contrasts in a voxel-wise manner including age and duration of education as covariates of no interest. Post-hoc *t*-tests between each pair of groups were masked with contrast of ANOVA across the three groups. All statistical analyses were corrected for multiple comparisons based on Monte Carlo simulations (3dClustSim, AFNI, http://afni.nimh.nih.gov/pub/dist/doc/program_help/3dClustSim.html). To be brief, 3dClustSim estimates the cluster size at an uncorrected p-value that is equivalent to corrected p-values by performing Monte Carlo simulations based on the smoothness of the data. The results from ANOVA were set at a combined individual *P* value of *P* < .005 and a cluster size of 30 voxels, with the threshold yielding a corrected *P* < .05. In order to correct for the total number of comparisons in the post-hoc *t*-tests, we applied a Bonferroni-like adjustment to the *t*-test significance level which resulted in a threshold of *P* < .005 with a cluster size of 40 voxels corresponding to *P* < .05/6.

### Clinical data analyses

To determine normal distribution of data, we first performed the Shapiro-Wilk test. Accordingly, means ± standard deviations were presented for data with normal distribution, while medians with interquartile ranges were presented for data not normally distributed. ANOVA was used to compare age which was normally distributed, while the Kruskal-Wallis Test was used to compare duration of education which was not normally distributed between the three groups. To compare the NP test results between the HAND and HIV-IC groups, the two-sample *t* test or Mann–Whitney *U* test and χ2 test or Fisher’s exact test was used to analyze quantitative and qualitative data, respectively.

To examine what cognitive performances were associated with altered intrinsic brain activities in the HAND, we carried out correlation analyses between brain regions and NP test results that showed significant differences in the HAND compared to the HIV-IC. We defined the regions-of-interest (ROIs) including the primary sensorimotor area and orbitofrontal cortex which showed significant differences between the HAND and HIV-IC in ReHo and fALFF analyses, respectively. Then, normalized values of each voxel in ROI were extracted and averaged for each individual map. As the values from ReHo and fALFF highly anti-correlated with each other, the differences between ReHo and fALFF values were also included in the analysis to examine how the interaction between these two regions were related to cognitive change in HAND. Thus, the mean values for each of the ROIs and the subtracted values of all HIV patients were correlated with their scores on the target NP tests using Pearson’s or Spearman correlation, whichever was appropriate. All statistical analyses were performed using the statistics toolbox of Matlab R2014a or SPSS version 24.0 (IBM, Armonk, New York). A two-tailed, Bonferroni-corrected *P* < .05 was considered statistically significant.

## Results

### Demographic characteristics and NP test results

Among the 11 patients with HAND, one patient was excluded due to progression of liver cirrhosis that might affect cognitive function. Of the 13 patients with HIV-IC, one was excluded due to significant substance use. Therefore, 10 patients with HAND, 12 with HIV-IC, and 11 HCs were included in this study. In the 10 patients with HAND, two had asymptomatic neurocognitive impairment and eight had mild neurocognitive impairment. No patient had HIV-associated dementia.

There were no significant differences between the groups (HCs, HIV-IC, HAND) with respect to age (53.0 ± 7.39, 52.5 ± 6.43, and 56.0 ± 8.15; *P* = .50) and duration of education (12.00 [12.00–12.00], 12.00 [12.00–16.00], and 13.25 [9.00–16.00]; *P* = .96). All HIV patients who were on HAART had undetectable plasma viral loads (< 20 copies/mL) except one patient with HAND whose plasma HIV RNA was also low (52.2 copies/mL). There was no significant difference in current CD4 cell count (704.33 ± 201.45 vs. 525.80 ± 252.24; *P* = .080) and disease duration defined as years since HIV diagnosis (9.5 [8.50–14.50] vs. 10.0 [4.75–39.00]; *P* = .668). Compared to HIV-IC, HAND showed decreased ability in learning and recall, abstraction/executive function, and sensory perception/motor skills domains (Table 1).

**Table 1:** Neuropsychological status of HIV-IC and HAND.

	HIV-IC (n = 12)	HAND (n = 10)	*P value*
**Speed of Information Processing (preserved/impaired)**^§^	12/0	9/1	0.455
K-WAIS Digit Symbol Subtest, ASS^¶^	13.00 (12.25–14.50)	13.00 (10.75–14.00)	0.587
TMT A, s^¶^	25.73 (20.70–30.76)	34.34 (30.00–52.53)	0.021
**Memory including Learning and Recall (preserved/impaired)**^§^	12/0	5/5	0.010
K-AVLT Delayed Recall, ASS	11.75 ± 2.14	7.9 ± 3.84	0.008
K-AVLT Delayed Recognition, ASS	11.75 ± 2.67	9.7 ± 3.20	0.117
K-AVLT Total (Trial 1–5), ASS/5	12.10 ± 1.38	9.56 ± 2.15	0.003
KCFT Copy, ASS^¶^	16.50 (13.25–17.00)	16.50 (14.50–17.00)	1.000
KCFT Immediate Recall, ASS^¶^	15.00 (12.50–16.75)	14.00 (10.75–16.00)	0.317
KCFT Delayed Recall, ASS^¶^	15.00 (12.50–16.75)	14.50 (11.00–15.50)	0.440
**Abstraction or Executive Function (preserved/impaired)**^§^	11/1	2/8	0.002
WCST Conceptual Level Responses, SS	103.00 ± 16.53	82.60 ± 14.49	0.006
WCST Perseverative Errors, SS	106.92 ± 20.01	87.80 ± 12.93	0.017
WCST Total Number of Errors, SS	103.75 ± 16.44	81.80 ± 15.97	0.005
K-WAIS Similarity Subtest, ASS	12.67 ± 2.06	12.20 ± 2.39	0.628
TMT B, s^¶^	79.10 (61.48–90.41)	144.86 (85.39–279.16)	0.030
**Attention or Working memory (preserved/impaired)**^§^	12/0	12/0	N/A
K-WAIS Digits Span Subtest, ASS	13.67 ± 2.77	11.90 ± 2.96	0.168
**Sensory Perception or Motor skills (preserved/impaired)**^§^	10/2	2/8	0.008
Grooved Pegboard Test, s^¶^	59.69 (57.02–68.92)	80.43 (67.31–98.28)	0.004
**Verbal or Language (preserved/impaired)**^§^	12/0	1/9	0.455
K-WAIS Vocabulary Subtest, ASS^¶^	12.00 (10.00–13.00)	11.00 (10.75–12.00)	0.331

^§^Data are numbers,

^¶^Data are medians (interquartile ranges), Unless otherwise indicated, data are mean values ± standard deviation.

ASS: age-corrected scaled scores (mean = 10, standard deviation = 3), K-AVLT: Korean Rey Auditory Verbal Learning Test, KCFT: Age-corrected scores on Korean Complex Figure Test, K-WAIS: Korean Wechsler Adult Intelligence Scale, N/A: not applicable, s: seconds, SS: age-corrected standardized scores (mean = 100, standard deviation = 15), WCST: Wisconsin Card Sorting Test.

### ReHo

**Fig 1:**
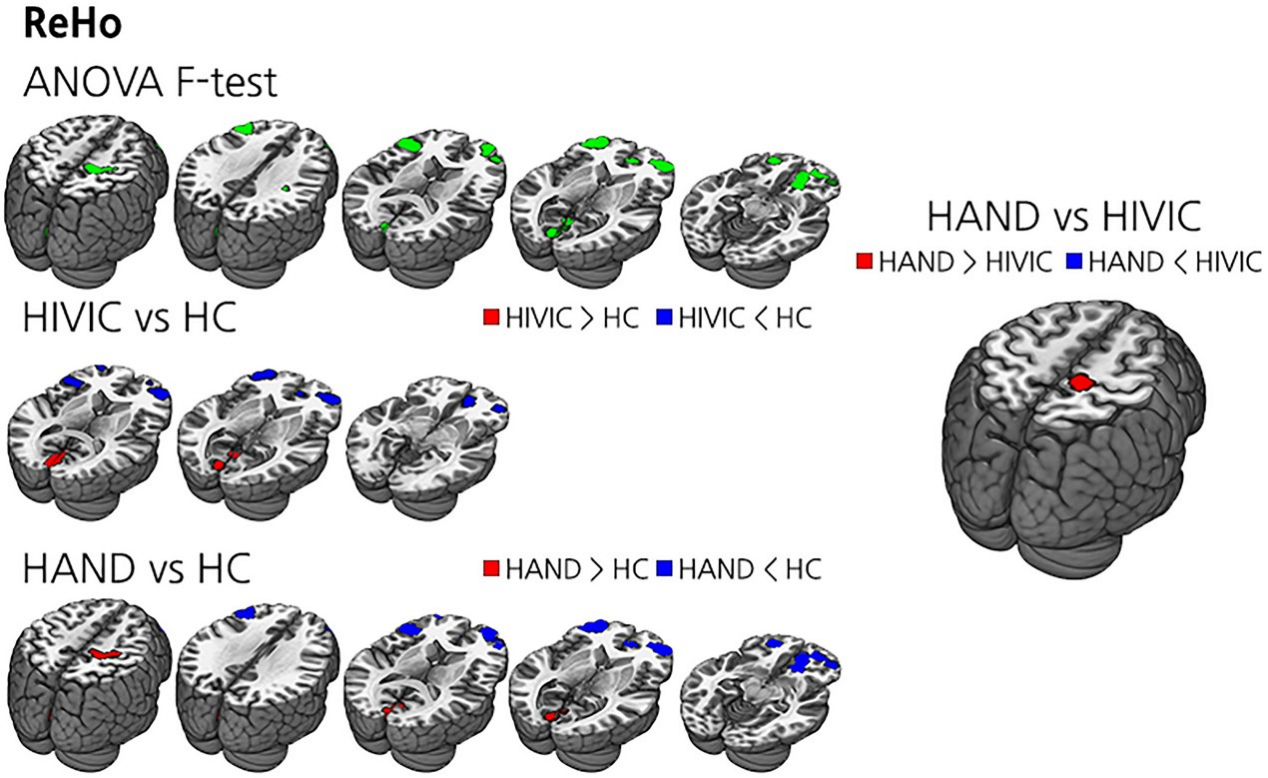
The results of ReHo analysis. (Top left) ANOVA results between HAND, HIV-IC and HC. Green clusters are voxels with significant ANOVA results. (Middle left) *t*-test results between HIV-IC and HC. Blue clusters represent lower ReHo regions of HIV-IC and red clusters represent the opposite. (Bottom left) *t*-test results between HAND and HC. Blue clusters represent lower ReHo regions of HAND and red clusters represent the opposite. (Right) *t*-test results between HAND and HIV-IC. Blue clusters represent lower ReHo regions of HAND and red clusters represent the opposite.

On ANOVA analysis, several clusters in the bilateral frontal, bilateral medial occipital, and the right sensorimotor areas showed significant differences between the groups. According to the post-hoc analysis, HIV-IC had lower ReHo in the bilateral frontal areas, while having higher ReHo in the medial occipital areas compared to HC. HAND showed broader areas showing decreased ReHo in the bilateral frontal areas and increased ReHo in the right primary sensorimotor area as well as the medial occipital areas compared to HC. In direct comparison with HIV-IC, HAND had higher ReHo in the right primary sensorimotor area, while there was no area showing lower ReHo in HAND (Table 2 and Fig 1).

**Table 2:** Regions showing significant differences in rsfMRI values between the groups.

Seed	Contrast	Region	Side	MNI Coordinates			Maximum *t*	No. of voxels	*P* value
				x	y	z			
ReHo	HIV-IC > HC	Lingual gyrus	R	3	-60	6	4.06	77	< 0.001
	HIV-IC < HC	Inferior frontal triangularis	L	-51	27	9	5.22	96	< 0.001
		Middle frontal gyrus	R	33	54	24	4.83	199	< 0.001
		Middle orbitofrontal gyrus	L	-42	48	-3	4.3	64	< 0.001
		Medial orbitofrontal gyrus	R	6	48	-9	3.63	54	0.001
	HAND > HC	Paracentral lobule	R	12	-30	60	5.18	78	< 0.001
		Calcarine	R	6	-72	12	3.72	64	< 0.001
	HAND < HC	Rectus gyrus	R	12	30	-15	5.94	159	< 0.001
		Middle orbitofrontal gyrus	L	-24	51	-12	5.27	135	< 0.001
		Inferior frontal triangularis	L	-51	30	9	5.1	108	< 0.001
		Middle orbitofrontal gyrus	R	36	54	-3	4.82	222	< 0.001
		Inferior frontal triangularis	L	-48	30	30	4.4	53	< 0.001
	HAND > HIV-IC	Paracentral lobule	R	15	-30	57	4.66	40	< 0.001
ALFF	HIV-IC > HC	Cuneus	R	6	-81	39	5.04	526	< 0.001
	HIV-IC < HC	Inferior orbitofrontal gyrus	L	-39	54	-12	5.37	243	< 0.001
		Middle frontal gyrus	R	24	30	39	5.32	45	< 0.001
		Insula	L	-30	18	12	5.26	65	< 0.001
		Medial orbitofrontal gyrus	L	-6	48	-12	4.29	46	< 0.001
		Superior temporal pole gyrus	L	-51	6	0	4.01	44	< 0.001
	HAND > HC	Cuneus	L	-9	-93	6	5.12	596	< 0.001
	HAND < HC	Middle orbitofrontal gyrus	L	-39	54	-12	5.92	280	< 0.001
		Precentral gyrus	L	-33	3	33	5.82	100	< 0.001
		Middle frontal gyrus	L	-21	21	45	4.99	95	< 0.001
		Insula	L	-30	18	12	4.94	63	< 0.001
		Superior temporal gyrus	L	-51	-9	-3	4.83	92	< 0.001
fALFF	HAND < HC	Middle orbitofrontal gyrus	R	36	45	-9	6.74	73	< 0.001
	HAND < HIV-IC	Middle orbitofrontal gyrus	R	39	45	-9	4.47	59	< 0.001

Note.—L: left, R: right

### ALFF

**Fig 2:**
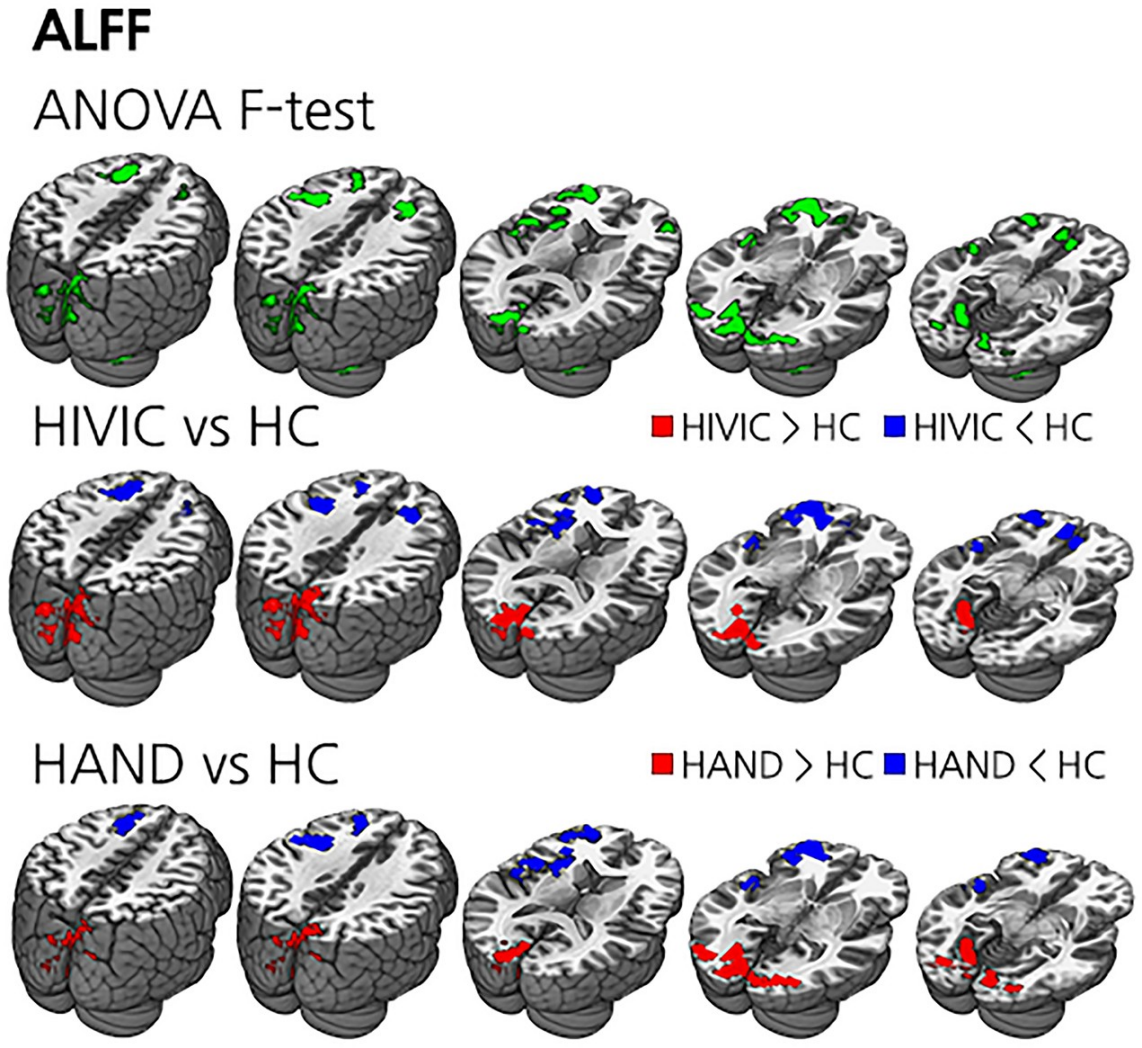
The results of ALFF analysis. (Top) ANOVA results between HAND, HIV-IC and HC. Green clusters are voxels with significant ANOVA results. (Middle) *t*-test results between HIV-IC and HC. Blue clusters represent lower ALFF regions of HIV-IC and red clusters represent the opposite. (Bottom) *t*-test results between HAND and HC. Blue clusters represent lower ALFF regions of HAND and red clusters represent the opposite. There was no significant voxel in comparison between HAND and HIV-IC.

ALFF also showed significant group difference in the bilateral frontal, temporal, and occipital areas on ANOVA analysis. Compared to HC, HIV-IC and HAND had lower ALFF in the bilateral frontal areas, while higher ALFF was observed in the bilateral occipital areas. No significant difference was found in ALFF between HAND and HIV-IC (Table 2 and Fig 2).

### fALFF

**Fig 3:**
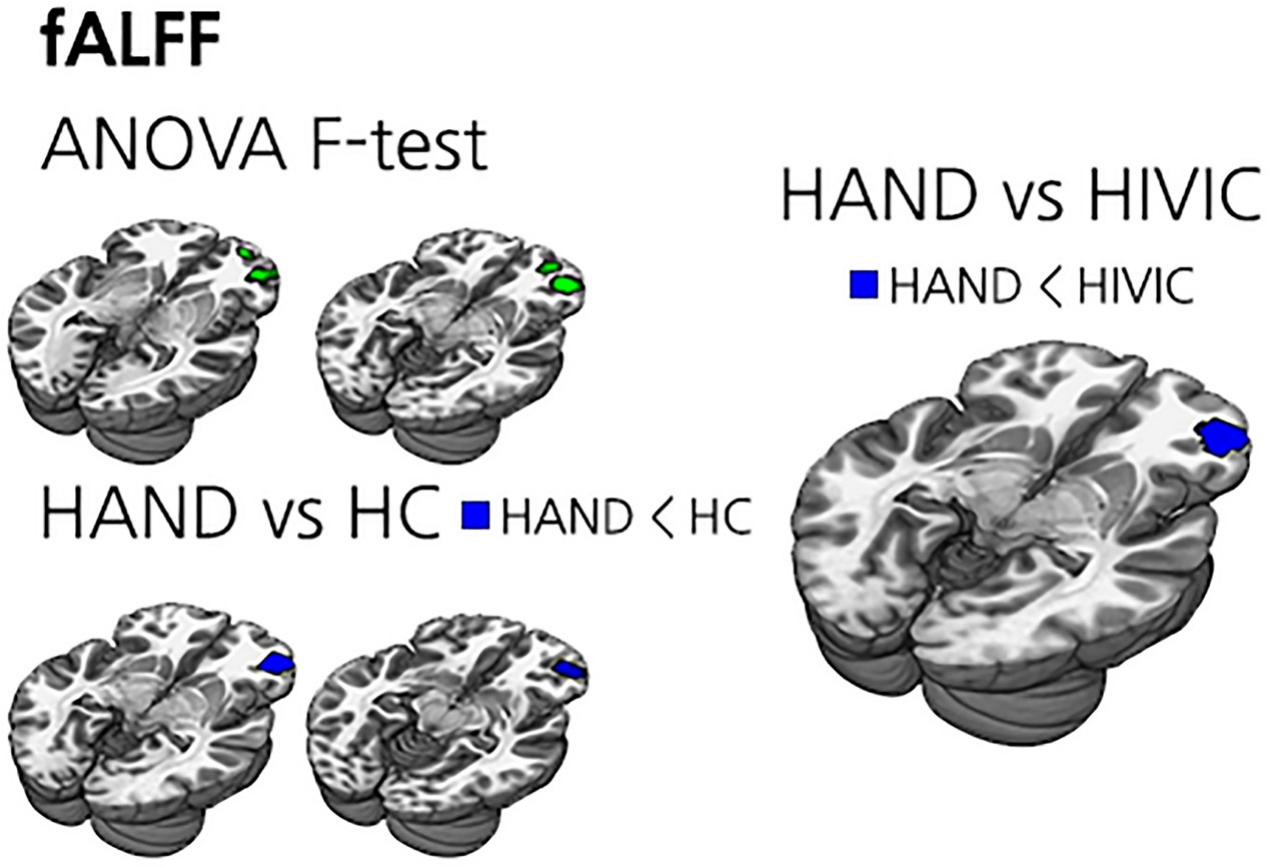
The results of fALFF analysis. (Top left) ANOVA results between HAND, HIV-IC and HC. Green clusters are voxels with significant ANOVA results. (Bottom left) *t*-test results between HAND and HC. Blue clusters represent lower ALFF regions of HAND, and there was no significant voxel for the opposite. (Right) *t*-test results between HAND and HIV-IC. Blue clusters represent lower ALFF regions of HAND, and there was no significant voxel for the opposite. No significant voxel was found in a comparison between HIV-IC and HC.

A few clusters in the right frontal areas were found to be significantly different in fALFF between groups on ANOVA analysis. HAND had lower fALFF in the right frontal areas compared to HIV-IC as well as HC. There was no significant difference in fALFF between HIV-IC and HC (Table 2 and Fig 3).

### Correlation analyses between rsfMRI values and NP test results

**Fig 4:**
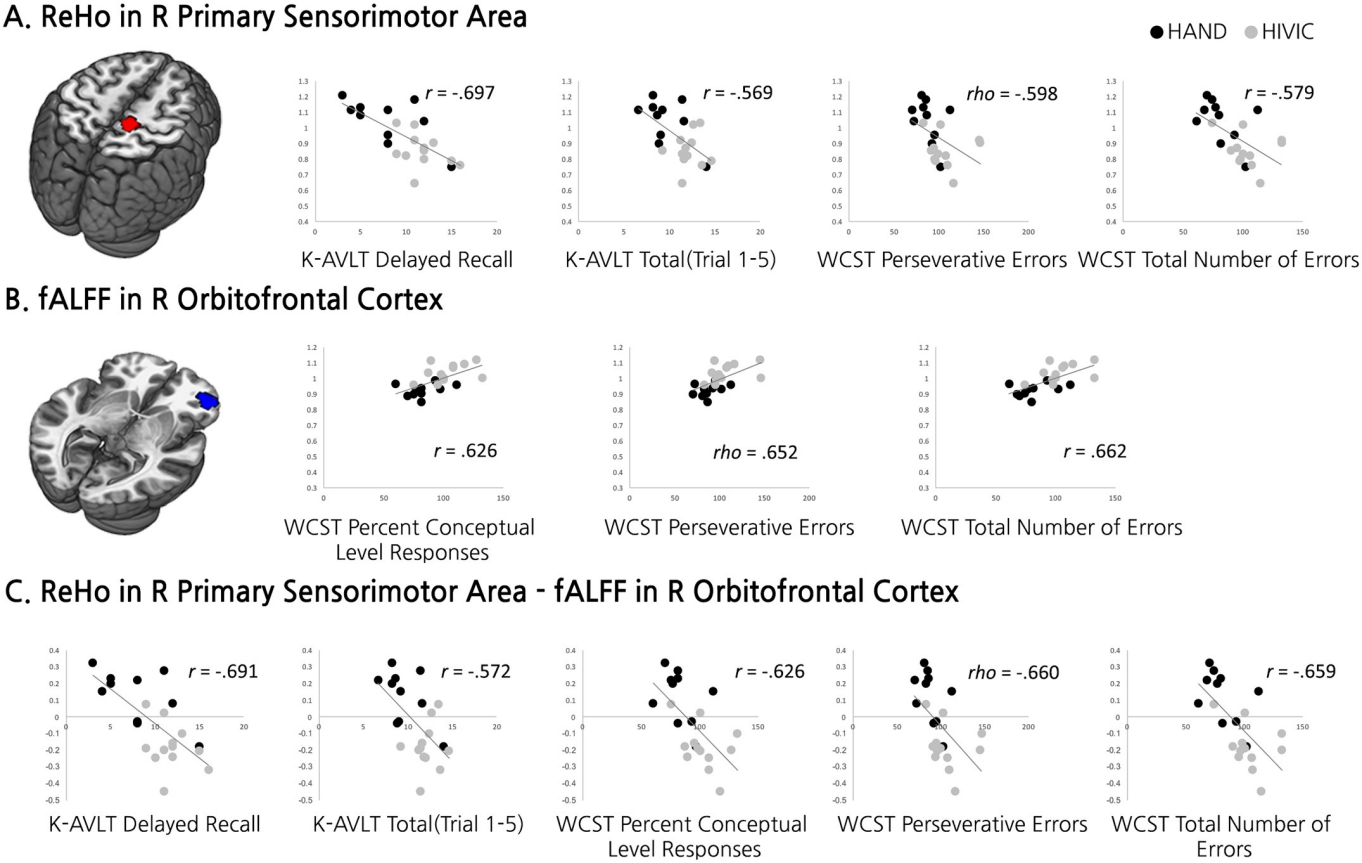
The results of correlation analysis between mean standardized rsfMRI values and NP test scores. (A) The left panel shows the ROI of the primary sensorimotor area which showed significant difference in ReHo values between HAND and HIV-IC. Scatter plots depicted in the right panel show significant correlations between mean ReHo values in the ROI and NP test scores. (B) The left panel shows the ROI of the orbitofrontal cortex which showed significant difference in fALFF values between HAND and HIV-IC. Scatter plots depicted in the right panel show significant correlations between mean fALFF values in the ROI and NP test scores. (C) Scatter plots show significant correlations between NP test scores and the rsfMRI values where standardized fALFF values in the orbitofrontal cortex are subtracted from standardized ReHo values in the primary sensorimotor area.

According to the group comparison results, we defined two ROIs in the right primary sensorimotor area (58 voxels) and right middle orbitofrontal area (40 voxels) which showed significant difference in ReHo and fALFF, respectively, between HAND and HIV-IC (Fig 4). Among the eight NP tests which showed significant group differences, poor performances in the delayed recall and total scores of K-AVLT and perseverative error number and total error number of WCST were significantly correlated with higher mean ReHo in the right primary sensorimotor area (*Ps* < 0.05/8). Poor performance in WCST subsets was also significantly correlated with lower mean fALFF in the right middle orbitofrontal area (*Ps* < 0.05/8). Finally, higher subtracted values between the primary sensorimotor area and orbitofrontal cortex were correlated with worse performance in all of the AVLT and WCST subsets (*Ps* < 0.05/8; Table 3).

**Table 3:** Correlation analysis of selected ROIs and cognitive function tests.

rsfMRI value	Regions of interests		TMT A, s	K-AVLT Delayed Recall, ASS	K-AVLT Total (Trial 1–5), ASS/5	WCST Conceptual Level Responses, SS	WCST Perseverative Errors, SS	WCST Total Number of Errors, SS	TMT B, s	Grooved Pegboard Test, s
ReHo	Right paracentral lobule	Correlation coefficient	0.35^¶^	-.697**	-.569**	-0.551	-.598^¶^	-0.579	0.344^¶^	0.319^¶^
		*P* value	0.111	<0.001**	0.006**	0.008	0.003**	0.005**	0.117	0.148
fALFF	Right middle orbitofrontal	Correlation coefficient	-.477^¶^	0.511	0.437	0.626	0.652^¶^	0.662	-.497^¶^	-.384^¶^
		*P* value	0.025	0.015	0.042	0.002**	0.001**	0.001**	0.018	0.078
ReHo—fALFF		Correlation coefficient	0.389^¶^	-.691	-.572	-.626	-.660^¶^	-.659	0.378^¶^	0.355^¶^
		*P* value	0.074	<0.001**	0.005**	0.002**	0.001**	0.001**	0.083	0.105

^¶^Data are Spearman’s rho.

Unless otherwise indicated, data are Pearson’s *r*, ASS: age-corrected scaled scores (mean = 10, standard deviation = 3), K-AVLT: Korean Rey Auditory Verbal Learning Test, s: seconds, SS: age-corrected standardized scores (mean = 100, standard deviation = 15), WCST: Wisconsin Card Sorting Test.

** *P* < 0.05/8

## Discussion

In the present study, patients with HIV exhibited alterations of spontaneous brain activity regardless of cognitive dysfunction, even though they had undetectable or very low plasma HIV RNA with HAART. The HAND and HIV-IC groups showed similar activity patterns: lower rsfMRI values mainly in the bilateral frontal areas, and higher values in the posterior brain areas compared to HC. When compared to the HIV-IC group, the HAND group showed lower fALLF in the frontal area and higher ReHo in the primary sensorimotor area, and these areas were correlated with cognitive function in HIV patients, suggesting that these areas are relevant to cognitive impairment in HIV patients.

In our study, patients with HAND showed decreased ability in memory and abstraction/executive function as well as motor skills compared to the HIV-IC group. Meanwhile, information processing speed, attention/working memory, and verbal fluency were preserved in the HAND group. This is in line with previous results that show the presence of more cortical involvement with memory/learning and executive dysfunction in the post-HAART era than in the pre-HAART era as subcortical involvement was predominant in the pre-HAART era with disturbed motor skills, cognitive speed, and verbal fluency in patients with HAND [21]. According to these findings, one can postulate that patients with HAND might have alterations predominantly in the frontal and parietal cortices in the post-HAART era, as these areas comprise the executive network that plays a significant role in regulating executive functions [22], the default mode network relevant to learning and memory function [23,24], and the salience network that plays a switching role between the two networks [25].

As assumed, previous neuroimaging studies [26,27] found an association between cognitive impairment and frontal and parietal cortical thinning which might be partly attributable to the loss of synapses and particular types of neurons and reduced dendritic complexity [28,29] in HIV patients. A previous study using fMRI [30] also reported altered brain activation in the fronto-parietal regions during an executive function task. In this past study, HIV patients with mild cognitive compromise showed frontal hypo-activation and parietal hyper-activation during the task. The authors thus suggested reduced efficiency in the frontal regions and the recruitment of additional posterior regions to compensate for frontal disruption. Through rsfMRI studies [6,9], the authors showed decreased connectivity in the default mode, executive, and salience networks in HIV patients with all or some patients with HAND compared to controls and associations with decreased cognitive performance. By directly comparing HIV patients with and without HAND, our colleagues found decreased connectivity between the PCC and frontal areas in the default mode network [8].

In our study, we found intrinsic functional alteration in the frontal and parieto-occipital areas in HIV patients regardless of cognitive status. The HAND group consistently showed decreased rsfMRI values in the frontal/insular area and elevated values in the posterior area which covered some areas of the executive, default mode, and salience networks, keeping in line with previous results [6,8,9]. Unexpectedly, in our study, the HIV-IC group also showed similar patterns of altered intrinsic brain activation with the HAND group. This functional alteration might proceed clinically overt cognitive decline which can be detected in neuropsychological tests. In other words, some subtle—clinically undetected—changes in the cognitive process might already be present even before HAND is diagnosed in HIV-infected patients. Future studies with more cognitive demanding tests are warranted to reveal early changes in cognition and their clinical implications for HIV patients.

Among the frontal and posterior regions, small cortical areas including the orbitofrontal cortex and primary sensorimotor area were particularly more altered in HAND compared to HIV-IC, suggesting a possible role of those areas in developing HAND in HIV patients. As we found altered activity in the frontal cortex but not in the PCC, the role of the frontal area between these two regions might be more important in the case of decreased functional connectivity between the PCC and frontal areas in HAND relative to HIV-IC, which was noted in a previous study [8].

The orbitofrontal cortex has connections with the ventral striatum involved in reward processing [31], so one important function of the orbitofrontal cortex is known as rapid updating of stimulus-reward association [32]. In line with this, our results showed that lower fALLF values in the orbitofrontal cortex were significantly correlated with increased perseverative number of errors in WCST. This suggests that patients with altered orbitofrontal activity at rest had difficulties in discarding previous strategies and updating new responses that receive positive feedback as a reward [33,34].

Motor-related brain areas have also been consistently associated with HAND in previous research [35–37], as reflected in the former name of the less severe form of HIV-associated dementia (i.e., minor cognitive motor disorder). Our results also showed that altered activations in the primary sensorimotor area in HAND relative to HIV-IC were correlated with verbal memory and executive function. Unexpectedly, however, the altered activation of the primary sensorimotor cortex was not correlated with fine motor function in HIV patients. Previous studies have reported that decreased fine motor function is attributable to cortical thinning [27] or altered brain activity measured with magnetoencephalography [38] in the higher-order brain regions including the frontal cortex, but not the primary sensorimotor cortex. Another study with magnetoencephalography also revealed compensatory hyper-activation in the frontal areas along with decreased activation in the primary motor cortex during motor tasks [39]. Therefore, fine motor function might be controlled by more complicated networks rather than just the primary sensorimotor cortex. Further investigation is required to reveal the underlying process.

More interestingly, the activation of the primary sensorimotor area was highly anti-correlated with that of the orbitofrontal cortex like a seesaw, which might corroborate the compensatory hypothesis. The orbitofrontal cortex has a direct connection with the somatosensory cortex and integrates sensory information supporting higher level cognition [40]. Thus, it is possible that orbitofrontal disruption made the integration process inefficient and more sensory information is required, which made the primary sensorimotor area hyper-activated. It is also noticeable that the degree of compensatory activation between the two regions were increased with decreasing performance in all sub-fields of verbal memory and executive function that showed significant differences between HAND and HIV-IC, although each region was correlated with only part of those sub-fields. These results imply that the degree of imbalance between the two regions can be a useful indicator of cognitive impairment in HIV patients.

Small sample size is a main limitation of this study that undermines the possible generalization of its results. However, we believe our study gives insight to understanding the underlying mechanism behind cognitive impairment in HIV patients and we believe its results can be used to build up a hypothesis that can be confirmed by a future prospective study with larger sample size.

In conclusion, regardless of cognitive status, altered local intrinsic activities in the frontal and posterior brain regions were consistently found in HIV patients. However, the frontal and primary sensorimotor areas were more disrupted in HAND relative to HIV-IC and as these alterations were significantly associated with memory and abstraction/executive function in HIV patients, they might be more relevant to cognitive impairment in this population.

## Supporting information

S1 File Assessment of cognitive status and neuropsychological test results in healthy controls. (DOCX)

S2 File Information per subject. (XLSX)

## References

[1] Snider WD, Simpson DM, Nielsen S, Gold JW, Metroka CE, Posner JB. Neurological complications of acquired immune deficiency syndrome: analysis of 50 patients. Ann Neurol. 1983;14: 403–418. 10.1002/ana.410140404 6314874 10.1002/ana.410140404

[2] Heaton RK, Clifford DB, Franklin DR Jr., Woods SP, Ake C, Vaida F, et al HIV-associated neurocognitive disorders persist in the era of potent antiretroviral therapy: CHARTER Study. Neurology. 2010;75: 2087–2096. 10.1212/WNL.0b013e318200d727 21135382 10.1212/WNL.0b013e318200d727 PMC2995535

[3] Spudich S, Gonzalez-Scarano F. HIV-1-related central nervous system disease: current issues in pathogenesis, diagnosis, and treatment. Cold Spring Harb Perspect Med. 2012;2: a007120 10.1101/cshperspect.a007120 22675662 10.1101/cshperspect.a007120 PMC3367536

[4] Dore GJ, McDonald A, Li Y, Kaldor JM, Brew BJ, National HIVSC. Marked improvement in survival following AIDS dementia complex in the era of highly active antiretroviral therapy. AIDS. 2003;17: 1539–1545. 12824792 10.1097/00002030-200307040-00015

[5] Ku NS, Lee Y, Ahn JY, Song JE, Kim MH, Kim SB, et al HIV-associated neurocognitive disorder in HIV-infected Koreans: the Korean NeuroAIDS Project. HIV Med. 2014;15: 470–477. 10.1111/hiv.12137 24580888 10.1111/hiv.12137 PMC4140963

[6] Thomas JB, Brier MR, Snyder AZ, Vaida FF, Ances BM. Pathways to neurodegeneration: effects of HIV and aging on resting-state functional connectivity. Neurology. 2013;80: 1186–1193. 10.1212/WNL.0b013e318288792b 23446675 10.1212/WNL.0b013e318288792b PMC3691785

[7] Wang X, Foryt P, Ochs R, Chung JH, Wu Y, Parrish T, et al Abnormalities in resting-state functional connectivity in early human immunodeficiency virus infection. Brain Connect. 2011;1: 207–217. 10.1089/brain.2011.0016 22433049 10.1089/brain.2011.0016 PMC3621309

[8] Ann HW, Jun S, Shin NY, Han S, Ahn JY, Ahn MY, et al Characteristics of Resting-State Functional Connectivity in HIV-Associated Neurocognitive Disorder. PLoS One. 2016;11: e0153493 10.1371/journal.pone.0153493 27104345 10.1371/journal.pone.0153493 PMC4841538

[9] Chaganti JR, Heinecke A, Gates TM, Moffat KJ, Brew BJ. Functional Connectivity in Virally Suppressed Patients with HIV-Associated Neurocognitive Disorder: A Resting-State Analysis. AJNR Am J Neuroradiol. 2017;38: 1623–1629. 10.3174/ajnr.A5246 28596187 10.3174/ajnr.A5246 PMC7960403

[10] Kendall M, Gibbons JD. Rank correlation Methods. 5th ed London: Edward Arnold; 1990.

[11] Zang Y, Jiang T, Lu Y, He Y, Tian L. Regional homogeneity approach to fMRI data analysis. Neuroimage. 2004;22: 394–400. 10.1016/j.neuroimage.2003.12.030 15110032 10.1016/j.neuroimage.2003.12.030

[12] Chao-Gan Y, Yu-Feng Z. DPARSF: A MATLAB Toolbox for "Pipeline" Data Analysis of Resting-State fMRI. Front Syst Neurosci. 2010;4: 13 10.3389/fnsys.2010.00013 20577591 10.3389/fnsys.2010.00013 PMC2889691

[13] Antinori A, Arendt G, Becker JT, Brew BJ, Byrd DA, Cherner M, et al Updated research nosology for HIV-associated neurocognitive disorders. Neurology. 2007;69: 1789–1799. 10.1212/01.WNL.0000287431.88658.8b 17914061 10.1212/01.WNL.0000287431.88658.8b PMC4472366

[14] Kim HG, Shin NY, Bak Y, Kim KR, Jung YC, Han K, et al Altered intrinsic brain activity after chemotherapy in patients with gastric cancer: A preliminary study. Eur Radiol. 2016 10.1007/s00330-016-4578-x 27878584 10.1007/s00330-016-4578-x

[15] Heaton RK, Chelune GJ, Talley JL, Kay GG, Curtiss G. Wisconsin Card Sorting Test Maunual, Revised and Expanded. Odessa, FL: Psychological Assessment Resources, Inc; 1993.

[16] Kim HK. Handbook of Rey-Kim Memory Assessment. Taegu, Korea: Neuropsychology Press; 1999.

[17] Kim M, Hyun MH. Relationships between Trail Making Test(A, B, B-A. B/A) Scores and Ape, Education, comparison of performance head injury patient and psychiatric patient. The Korean Journal of Clinical Psychology. 2004;23: 323–366.

[18] Lee TY. Normative values for the Grooved Pegboard Test in Adult. Physical Therapy Korea. 2001;8: 87–94.

[19] Yeom TH, Park YS, Oh KJ, Lee YH. Korean Version Wechsler Adult Intelligence Scale. Seoul: Korea Guidance; 1992.

[20] Zou QH, Zhu CZ, Yang Y, Zuo XN, Long XY, Cao QJ, et al An improved approach to detection of amplitude of low-frequency fluctuation (ALFF) for resting-state fMRI: fractional ALFF. J Neurosci Methods. 2008;172: 137–141. 10.1016/j.jneumeth.2008.04.012 18501969 10.1016/j.jneumeth.2008.04.012 PMC3902859

[21] Heaton RK, Franklin DR, Ellis RJ, McCutchan JA, Letendre SL, Leblanc S, et al HIV-associated neurocognitive disorders before and during the era of combination antiretroviral therapy: differences in rates, nature, and predictors. J Neurovirol. 2011;17: 3–16. 10.1007/s13365-010-0006-1 21174240 10.1007/s13365-010-0006-1 PMC3032197

[22] Kane MJ, Engle RW. The role of prefrontal cortex in working-memory capacity, executive attention, and general fluid intelligence: an individual-differences perspective. Psychon Bull Rev. 2002;9: 637–671. 12613671 10.3758/bf03196323

[23] Herting MM, Nagel BJ. Differences in brain activity during a verbal associative memory encoding task in high- and low-fit adolescents. J Cogn Neurosci. 2013;25: 595–612. 10.1162/jocn_a_00344 23249350 10.1162/jocn_a_00344 PMC3786681

[24] Elman JA, Rosner ZA, Cohn-Sheehy BI, Cerreta AG, Shimamura AP. Dynamic changes in parietal activation during encoding: implications for human learning and memory. Neuroimage. 2013;82: 44–52. 10.1016/j.neuroimage.2013.05.113 23732887 10.1016/j.neuroimage.2013.05.113

[25] Goulden N, Khusnulina A, Davis NJ, Bracewell RM, Bokde AL, McNulty JP, et al The salience network is responsible for switching between the default mode network and the central executive network: replication from DCM. Neuroimage. 2014;99: 180–190. 10.1016/j.neuroimage.2014.05.052 24862074 10.1016/j.neuroimage.2014.05.052

[26] Thompson PM, Dutton RA, Hayashi KM, Toga AW, Lopez OL, Aizenstein HJ, et al Thinning of the cerebral cortex visualized in HIV/AIDS reflects CD4+ T lymphocyte decline. Proc Natl Acad Sci U S A. 2005;102: 15647–15652. 10.1073/pnas.0502548102 16227428 10.1073/pnas.0502548102 PMC1266080

[27] Shin NY, Hong J, Choi JY, Lee SK, Lim SM, Yoon U. Retrosplenial cortical thinning as a possible major contributor for cognitive impairment in HIV patients. Eur Radiol. 2017;27: 4721–4729. 10.1007/s00330-017-4836-6 28409354 10.1007/s00330-017-4836-6

[28] Wiley CA, Masliah E, Morey M, Lemere C, DeTeresa R, Grafe M, et al Neocortical damage during HIV infection. Ann Neurol. 1991;29: 651–657. 10.1002/ana.410290613 1909852 10.1002/ana.410290613

[29] Everall I, Luthert P, Lantos P. A review of neuronal damage in human immunodeficiency virus infection: its assessment, possible mechanism and relationship to dementia. J Neuropathol Exp Neurol. 1993;52: 561–566. 8229074 10.1097/00005072-199311000-00002

[30] Melrose RJ, Tinaz S, Castelo JM, Courtney MG, Stern CE. Compromised fronto-striatal functioning in HIV: an fMRI investigation of semantic event sequencing. Behav Brain Res. 2008;188: 337–347. 10.1016/j.bbr.2007.11.021 18242723 10.1016/j.bbr.2007.11.021

[31] Schultz W, Apicella P, Scarnati E, Ljungberg T. Neuronal activity in monkey ventral striatum related to the expectation of reward. J Neurosci. 1992;12: 4595–4610. 1464759 10.1523/JNEUROSCI.12-12-04595.1992 PMC6575755

[32] Rolls ET. Emotion and Decision-making Explained: Oxford University Press; 2013.

[33] Rolls ET, Hornak J, Wade D, McGrath J. Emotion-related learning in patients with social and emotional changes associated with frontal lobe damage. J Neurol Neurosurg Psychiatry. 1994;57: 1518–1524. 7798983 10.1136/jnnp.57.12.1518 PMC1073235

[34] Buckley MJ, Mansouri FA, Hoda H, Mahboubi M, Browning PG, Kwok SC, et al Dissociable components of rule-guided behavior depend on distinct medial and prefrontal regions. Science. 2009;325: 52–58. 10.1126/science.1172377 19574382 10.1126/science.1172377

[35] Kallianpur KJ, Kirk GR, Sailasuta N, Valcour V, Shiramizu B, Nakamoto BK, et al Regional cortical thinning associated with detectable levels of HIV DNA. Cereb Cortex. 2012;22: 2065–2075. 10.1093/cercor/bhr285 22016479 10.1093/cercor/bhr285 PMC3412442

[36] Zhou Y, Li R, Wang X, Miao H, Wei Y, Ali R, et al Motor-related brain abnormalities in HIV-infected patients: a multimodal MRI study. Neuroradiology. 2017;59: 1133–1142. 10.1007/s00234-017-1912-1 28889255 10.1007/s00234-017-1912-1

[37] Becker KM, Heinrichs-Graham E, Fox HS, Robertson KR, Sandkovsky U, O'Neill J, et al Decreased MEG beta oscillations in HIV-infected older adults during the resting state. J Neurovirol. 2013;19: 586–594. 10.1007/s13365-013-0220-8 24297500 10.1007/s13365-013-0220-8 PMC3913174

[38] Wilson TW, Heinrichs-Graham E, Becker KM, Aloi J, Robertson KR, Sandkovsky U, et al Multimodal neuroimaging evidence of alterations in cortical structure and function in HIV-infected older adults. Hum Brain Mapp. 2015;36: 897–910. 10.1002/hbm.22674 25376125 10.1002/hbm.22674 PMC4491915

[39] Wilson TW, Heinrichs-Graham E, Robertson KR, Sandkovsky U, O'Neill J, Knott NL, et al Functional brain abnormalities during finger-tapping in HIV-infected older adults: a magnetoencephalography study. J Neuroimmune Pharmacol. 2013;8: 965–974. 10.1007/s11481-013-9477-1 23749418 10.1007/s11481-013-9477-1 PMC3809128

[40] Rolls ET. Convergence of sensory systems in the orbitofrontal cortex in primates and brain design for emotion. Anat Rec A Discov Mol Cell Evol Biol. 2004;281: 1212–1225. 10.1002/ar.a.20126 15470678 10.1002/ar.a.20126

