# Supplementary material for: Altered intrinsic local activity and cognitive dysfunction in HIV patients: A resting-state fMRI study
Source: PLoS One. 2018 Nov 29;13(11):e0207146. doi: 10.1371/journal.pone.0207146 (PMC6264476; doi:10.1371/journal.pone.0207146)
Supplement: S1 File — (DOCX) [file pone.0207146.s001.docx]

**Assessment of cognitive status in healthy controls**

Eleven male subjects without any clinical cognitive impairment or active neurological disorders who were recruited as controls for a different study [1] were also included as controls for this study. They were assessed in four cognitive domains using a set of neuropsychological (NP) tests: (1) verbal and performance intelligence [Korean version of Wechsler Adult Intelligence Scale (K-WAIS) Vocabulary subtest and Block Design subtest]; (2) memory (Rey-Kim Memory Test); (3) attention (K-WAIS digit span subtest and Wechsler Memory Scale-Ⅲ Spatial Span subtest); and (4) executive function (Stroop Test). Scores for the NP tests were expressed as either age-corrected scaled scores (ASS; mean = 10, standard deviation [SD] = 3), scaled scores (SS; mean = 10, SD = 3), or percentile ranks for raw scores.

Table S1. Neuropsychological test results in healthy controls

| Assessment of cognitive status | Control |
| --- | --- |
|  |  |
| K-WAIS Vocabulary subtest, ASS | 11.2±2.7 |
| K-WAIS Block design subtest, ASS | 12.3±2.5 |
| AVLT Total of 1~5, SS/5 | 12.0±1.5 |
| AVLT delayed recall, ASS | 11.6±1.4 |
| AVLT delayed recognition, ASS | 12.7±2.2 |
| KCFT copy, ASS | 13.9±2.0 |
| KCFT immediate recall, ASS | 13.4±3.3 |
| KCFT delayed recall, ASS | 13.0±3.4 |
| Rey-Kim MQ | 109.3±10.3 |
| Digit span forward, ASS | 12.6±3.0 |
| Digit span backward, ASS | 11.6±2.3 |
| Digit span sequencing, ASS | 10.5±2.3 |
| Digit span total, ASS | 12.0±1.9 |
| Spatial span forward, percentile | 44.2±29.5 |
| Spatial span backward, percentile | 67.6±25.6 |
| STROOP, percentile | 91.6±18.6 |

ASS; age-corrected scaled scores (mean = 10, SD = 3), AVLT; Auditory Verbal Learning Test, KCFT; K-Complex Figure Test, SS; scaled score (mean = 10, SD = 3), Rey-Kim MQ; and Rey–Kim Memory Quotient (mean = 100, SD = 15).

**Reference**

1. Kim HG, Shin NY, Bak Y, Kim KR, Jung YC, Han K, et al. Altered intrinsic brain activity after chemotherapy in patients with gastric cancer: A preliminary study. Eur Radiol. 2016. doi: 10.1007/s00330-016-4578-x.
